# Blinding in electric current stimulation in subacute neglect patients with current densities of 0.8 A/m^2^: a cross-over pilot study

**DOI:** 10.1186/s13104-020-05421-7

**Published:** 2021-01-25

**Authors:** Anna Gorsler, Ulrike Grittner, Nadine Külzow, Torsten Rackoll

**Affiliations:** 1 Kliniken Beelitz GmbH, Neurologische Rehabilitationsklinik, Paracelsusring 6a, 14547 Beelitz-Heilstätten, Germany; 2 Centre for Stroke Research Berlin, Charité–Universitätsmedizin Berlin, Berlin, Germany; 3 QUEST Center for Transforming Biomedical Research, Charité–Universitätsmedizin Berlin, Berlin, Germany; 4 Institute of Biometry and Clinical Epidemiology, Charité–Universitätsmedizin Berlin, Berlin, Germany; 5 Berlin Institute of Health, Berlin, Germany

**Keywords:** Visuospatial neglect, Stroke, Transcranial direct current stimulation, Blinding procedure, Pilot study

## Abstract

**Objective:**

Neglect after stroke is a disabling disorder and its rehabilitation is a major challenge. Transcranial direct current stimulation (tDCS) seems to be a promising adjuvant technique to improve standard care neglect therapy. Since electric fields are influenced by age-related factors, higher current densities are probably needed for effective treatment in aged stroke patients. Validation of treatment efficacy requires sham-controlled experiments, but increased current densities might comprise blinding. Therefore, a pilot study was conducted to test sham adequacy when using current density of 0.8 A/m^2^. Whether especially neglect patients who mainly suffer from perceptual and attentional deficits are able to differentiate beyond chance active from sham tDCS was investigated in a randomized cross-over design (active/sham stimulation) in 12 early subacute patients with left-sided hemineglect. Stimulation (0.8 A/m^2^) was performed simultaneous to standard care neglect therapy.

**Results:**

Odds ratio of correct guessing an atDCS condition compared to wrongly judge an atDCS condition as sham was 10.00 (95%CI 0.65–154.40, p = 0.099). However, given the small sample size and high OR, although likely somewhat overestimated, results require careful interpretation and blinding success in neglect studies with current densities of 0.8 A/m^2^ should be further confirmed.

## Introduction

Large right hemispheric stroke often results in a multimodal neglect [1, 2]. Parietal or fronto-temporo-parietal networks are frequently affected, which also compromise multi-sensory integration areas, and are linked to attentional and perceptual deficits [3]. Despite symptoms persist for more than a year in 30–40% of patients, and the poor prognostic outcome of neglect [4], only few therapies have been established in clinical practice [5]. Interventions targeting transcranial direct current stimulation (tDCS) might be a promising approach [6], but further research is necessary.

tDCS modulates cortical excitability and network strengthening and suppression [7–10], but it’s effectiveness could be influenced by numerous parameters such as the stimulation site, duration and current density (in A/m^2^) [11–13], or interindividual heterogenenity [14]. According to head modelling simulation studies [15] electric fields can be affected by different tissue types in the brain. Considering the age-related natural and lesion induced loss of brain tissue [16] higher current densities may be necessary to observe clinically relevant effects in patients [17, 18].

Evaluation of new treatment strategies requires double-blind sham-controlled studies. tDCS, even if mild, can evoke sensations (itching, burning), and may accordingly compromise blinding, particularly at higher currents [19, 20]. Despite age-related altered processing of perceptual or sensory information [21] robustness of blinding has been mainly tested in healthy young subjects with conflicting results [22–24]. Nevertheless, whether results even obtained in healthy older adults [24] apply to brain-damaged neglect patients, who are mainly suffer from perceptual and attentional deficits, remain unknown. So far, different current densities (0.29–0.8 A/m^2^) have been administered in neglect patients [25, 26]. Methodological heterogeneity and/or no information about controlling effective blinding challenge conclusive remarks. Therefore, the aim of the present pilot study was to investigate whether neglect patients can discern active (atDCS) and sham stimulation (stDCS) beyond chance using high current densities of 0.8 A/m^2^ to assure adequacy of sham procedure before it is applied in larger trials.

## Main text

### Methods

A cross-over, double-blinded intervention pilot study was performed to assess the feasibility of blinding procedure. Secondary, long-term changes in visuospatial neglect (VSN) symptoms were monitored during inpatient rehabilitation to gain information about recovery-rate in patients with first-time ever stroke in rehabilitative context for future trials. This pilot study closely agrees with CONSORT-Guidelines (Additional file 1).

The study was conducted within the neurological rehabilitation ward of the Kliniken Beelitz GmbH in Brandenburg, Germany. All patients were pre-screened for eligibility. Inclusion criteria comprised: ischemic or hemorrhagic stroke within the right hemisphere (confirmed by neuro-imaging), early subacute phase (> 7 and < 56 days after stroke onset), age ≥ 18 years, right-handed [27], and residual VSN symptoms. Major exclusion criteria included: history of stroke, severe cognitive impairment, epilepsy and the presence of a pacemaker (Additional file 2: Table S1).

#### Procedures

The presence of VSN was tested at screening visit using selected tests from the Behavioral Inattention Test battery (BIT, German version: Star Cancellation, Figure Copying, and Line Bisection [28]). Only patients with impaired performance in at least two of these tests, and confirmed VSN diagnosis by the treating neuropsychologist entered the baseline visit, which was scheduled approximately one week after the screening visit to account for spontaneous recovery of VSN symptoms. Subsequently, an atDCS and stDCS session were applied in randomized order (48 h wash-out period in between) during standard care neuropsychological therapy (30 min, exploration tasks) by the treating therapist. On the last day of the hospital stay patients were re-assessed (follow-up).

The randomization list was generated by a self-written script (Additional file 3) using R-statistical software (random generator). A stimulation protocol for atDCS and stDCS were programmed and performed by the same assessor (TR). Patients and treating therapists were blinded to the stimulation protocol.

tDCS was applied by a StarStim tDCS stimulator (Neuroelectrics, Barcelona, Spain) via electrodes (round electrodes, 25 cm^2^) mounted over both posterior parietal cortices (P4-anode; P3-cathode, bi-hemispheric protocol) determined with the international 10–20 EEG System with an intensity of 2 mA (current density: 0.8 A/m^2^). tDCS was delivered for 20 min (atDCS) or 30 s (stDCS) in a ramp-like fashion with a 15 s (fade in/fade out) interval at the beginning and the end of the stimulation.

#### Assessments

After each tDCS session patients were ask: “Do you think you received an active or sham stimulation or are you undecided?” to assess blinding success, and for the sensation of itching, pain, burning, heat, taste of metal, or fatigue during stimulation. Adverse events were monitored throughout the hospital stay and noted if they could be related to the intervention.

At baseline demographic and clinical data were recorded including impairment caused by stroke using the National Institute of Stroke Scale (NIHSS) [29]. Global cognitive functioning were assessed by the Montreal Cognitive Assessment (MoCA) [30]. VSN symptoms were assessed at baseline and follow-up (Star-, Letter-, and Line Cancellation, Line Bisection, Figure Copying and Text Reading [28]).

#### Statistical analysis

Twelve patients were included. Around 6% of all stroke patients admitted to the clinic were eligible patients for this study.

Each patient evaluated stimulation mode twice for stDCS and atDCS. Guessing answers of stimulation mode were coded as: (a) sham, (b) indifferent, (c) active. Binary logistic mixed models were applied to estimate if guessing of the stimulation condition was associated with atDCS-stimulation condition by accounting for the clustered data structure (repeated measures, random intercept model) (melogit command in stata). Patients judgements were included as independent (nominal), the actual stimulation condition as dependent variable (coded: atDCS: 1, stDCS: 0).

All analyses were performed in an exploratory framework with descriptive statistics presenting mean (SD) or median [IQR] depending on the distribution of the data. Sensations were aggregated into a new dichotomous variable “any sensation” (present /absent). Proportions of patients who perceived any sensation under atDCS and stDCS were compared by non-parametric McNemar test. Changes between performance in baseline and follow-up were analysed by Wilcoxon signed rank test. Cohen’s d with confidence intervals (CI) are reported as effect size. Analyses were not corrected for multiple testing. Data were analyzed by R- (Version 3.4.4 [31]) or Stata Statistical Software, Release 15 [32].

### Results

Between July 2018 and February 2019 686 patients were screened for eligibility. Twelve patients (3%) met all inclusion and exclusion criteria and gave written consent to participate. Three patients could not be assessed at follow-up due to early discharge (n = 2) or severe progression of visual impairment (Additional file 2: Figure S1).

On average, 26 [16–46] days elapsed since stroke. Between screening and baseline visit were [3] days without signs of spontaneous recovery. Patients (7 females) were between 65 and 83 years old (median 77). Median NIHSS was 7 [2–10]. Three patients showed signs of anosognosia and two patients were later suspected to have hemianopia. In all but Line Cancellation test, patients showed impaired performance in neglect tests at baseline (Table 1).

**Table 1:** Baseline characteristics and assessment of neglect symptoms at follow-up visit

	Baseline N = 12	Follow-Up N = 9 ¶	p-value	Effect size, CI^a^
Age in years, median [IQR]	77 [68–83]	–		
Female sex, n (%)	7 (58)	–		
Ischemic stroke, n (%)	9 (75)	–		
Time from stroke in days, median [IQR]	26 [16–46]	–		
NIHSS at inclusion, median [IQR]	7 [2–10]	–		
MoCA sum score, mean (SD)	18 (5)	20 (6)^b^	0.12	0.2 [− 0.1 to 0.5]
Star cancellation test, mean (SD)	32 (13)	41 (16)	0.65	0.2 [− 0.6 to 0.9]
Letter cancellation test, mean (SD)	25 (7)	32 (9)	0.13	0.6 [− 0.2 to 1.4]
Line cancellation test, median [IQR]	35 [28–36]	36 [34–36]	0.46	0.37 [− 0.4 to 1.1]
Line bisection test in cm, mean (SD)	2.6 (2)	0.5 (1.2)	0.07	1.0 [− 0.2 to 2.1]
Figure copying test, median [IQR]	3 [2–4]	6 [4–7]	0.1	0.5 [0.0 to 1.1]
Text reading test, median [IQR]	90 [86–133]	117 [87–136]	0.83	0.2 [− 0.5 to 0.8]

Data of one patient could not be analysed due to bad performance of patient

^a^Effect sizes are calculated using Cohen’s d

^b^One test could not be rated due to bad performance of the patient

Each patient evaluated both conditions (atDCS and stDCS) resulting in a total of 24 judges. Four out of twelve times the atDCS and five out of twelve times the stDCS protocol were identified correctly. Twelve times out of 24 ratings patients were indifferent, six times when evaluating stDCS and six times when evaluating the at DCS protocol (Table 2). Marginal probabilities for having an atDCS condition if guessed correctly was 80.0% (95% CI 30.9–97.3%). If sham was guessed the marginal probability of actually having an atDCS condition (wrong guessing) was 28.6% (95% CI 7.2–67.3%). If the judgement was indifferent the marginal probability of having an atDCS condition was 50.0% (95% CI 24.4–75.6%). The odds ratio of correct guessing an atDCS condition compared to wrongly judge an atDCS condition as sham was 10.00 (95% CI 0.65–154.40, p = 0.099). Wash-out phase between intervention sessions was 3 (2) days.

**Table 2:**
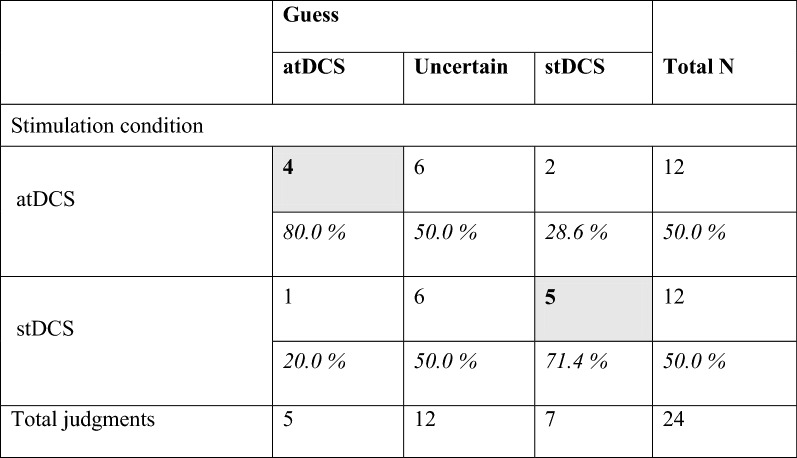
Distribution of guessing between active and sham stimulation among patients

Data is given as number of patients. Percentage is related to column-sum

Grey fields indicate correct guesses. *atDCS*  active transcranial direct current stimulation, *stDCS* sham transcranial direct current stimulation

After atDCS four and after stDCS one out of 12 patients reported the presence of any sensations, but marginal proportions were not substantially different (p = 0.25). Specifically, patients reported three times sensations of itching (during atDCS) and of burning (one during stDCS, two during atDCS), and one time the sensation of heat (during atDCS). In two of the seven cases patients correctly guessed atDCS stimulation. The remaining 5 patients stated “no idea” when asked about received stimulation condition (Table 3). No other adverse events occurred after intervention or during the study period.

**Table 3:**
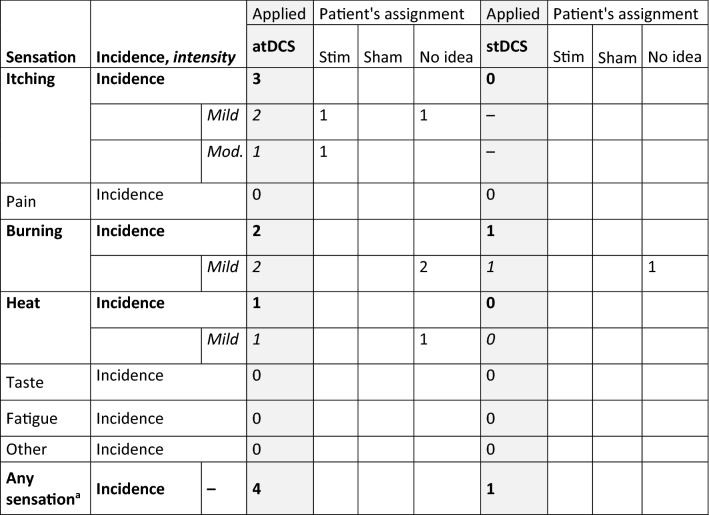
Frequency and intensity of sensations reported after active and sham tDCS stimulation and patient’s subjective assignment

Incidence is given as number of patients who reported a sensation. If patients perceived a sensation intensity as well as subjective assignment to tDCS condition is also reported. Intensity was measured on a self-rating scale (“no”, „mild “, „moderate “, „strong “) and is given as frequency of patients who reported this sensation. atDCS = active transcranial direct current stimulation; stDCS = sham transcranial direct current stimulation, Stim-active tDCS, sham-sham tDCS, mod.-moderate. ^a^Proportions of patients who reported any sensations between atDCS and stDCS were compared by McNemar Test. Marginal proportions were not substantially different from each other (p = .25). Perceived sensations are in bold

Mean difference between baseline and follow-up was 40 (26) days. No significant improvement in performance from baseline to follow-up was revealed, but a large effect size in the Line Bisection test was observed (Cohen’s d = 1.0, 95% CI − 0.2 to 2.1) (Table 1).

### Discussion

Considering that reliable blinding plays a pivotal role in placebo-controlled clinical trials, and higher current densities are probably needed to observe clinically relevant effects in brain damaged subjects, this study aimed to assess the effectiveness of blinding of an atDCS versus stDCS stimulation protocol applying a high current density (0.8 A/m). In the present pilot study neglect patients could not reliably distinguish between active and sham stimulation. Although analysis of OR did not reveal a statistically significant effect, analysis resulted in a close-to-significance p-value, and, descriptively in a high magnitude of correct guessing an atDCS condition compared to wrongly judge an atDCS condition as sham stimulation (OR = 10, p = 0.09). Given the small sample size and high rate of indifferent responses „no idea “ (50%) among judgements, OR could certainly have been somewhat overestimated. However, considering the pilot character of the study the preliminary provided evidence of blinding adequacy should be interpreted with caution and confirmed in further studies.

Despite the used high current density only few sensations of mild intensity were reported by patients, mostly itching and burning as in other studies [33, 34]. Because stimulation mode assignment (when a sensation has been perceived) was rather uncertain, it is unlikely that these sensations could fully account for the measured OR. Previous studies using comparable stimulation intensity (2 mA, 20 min/30 min), but lower current density (0.57 A/m^2^), could demonstrate successful blinding in young [23] and old [24] healthy subjects in a cross-over design, but they have also found that subjects tended to identify stimulation mode more correctly in the second of two sessions. This result was probably due to increased experience with tDCS in a within-subject design and might also explain the observed higher odds of correctly identifying the atDCS condition in our study. It should be noted that experience need not be limited to sensations caused by tDCS itself, but may also include any subjective changes (e.g., performance, symptom reduction), and this may be particularly relevant in the clinical population [35]. However, age-related differences in sensation and perception [21], and complaints about it [36] render a translation of results obtained in healthy (young) to clinical (brain-damaged) subjects difficult. Although in one study chronic stroke patients suffering from motor dysfunction did not differ in discomfort or stimulus identification from healthy controls [37], conclusions are limited considering marked deficits in neglect patients (perception, attention), differences in stimulus parameters (1 mA, 0.4 A/m^2^ vs. 2 mA, 0.8 A/m^2^), and phase (chronic vs. subacute). Overall, results may indicate that blinding could be more a concern of cross-over designs. However, caution is required when interpreting, evaluating, and comparing robustness of blinding in different studies. In addition to a number of study-related parameters (intensity, ramping, design), individual characteristics, age-related alterations, or specifics of the population under study must be considered. Since systematic research is lacking, strategies to prevent and control blinding efficacy across studies remain strongly recommended to improve our understanding about successful blinding procedures.

Recovery of neglect symptoms (secondary outcome) was limited to Line Bisection. Although this test is not specific to VSN [38], it was the most change-sensitive test in the present study. However, heterogeneous symptoms presented in VSN are associated with different cognitive demands and dissociations between tests are frequently observed [39]. The additional use of computer-based tests in future studies might provide further information about subtle changes in neglect symptoms [40].

## Limitations

- Small sample size
- Single-session intervention
- No assessment of blinding success of rater

## Supplementary Information

**Additional file 1.** Consort 2010 checklist.

**Additional file 2: Figure S1.** Flowchart of cross-over study. **Table S1.** Inclusion and Exclusion criteria.

**Additional file 3.** Randomization procedure.

## Data Availability

Data and analysis scripts are freely available for researchers on request to reproduce current findings or for further analysis.

## Abbreviations

atDCS: Active transcranial direct current stimulation
stDCS: Sham transcranial direct current stimulation
VSN: Visuospatial neglect
BIT: Behavioral Inattention Test battery
NIHSS: National Institute of Stroke Scale
MoCA: Montreal Cognitive Assessment
SD: Standard deviation
IQR: Interquartile range
CI: Confidence interval
CT: Computer tomography
MRI: Magnetic resonance imaging
