# Supplementary material for: Blinding in electric current stimulation in subacute neglect patients with current densities of 0.8 A/m2: a cross-over pilot study
Source: BMC Res Notes. 2021 Jan 25;14:35. doi: 10.1186/s13104-020-05421-7 (PMC7836170; doi:10.1186/s13104-020-05421-7)
Supplement: Supplementary file 2 — Additional file 2: Figure S1. Flowchart of cross-over study. Table S1. Inclusion and Exclusion criteria. [file 13104_2020_5421_MOESM2_ESM.docx]

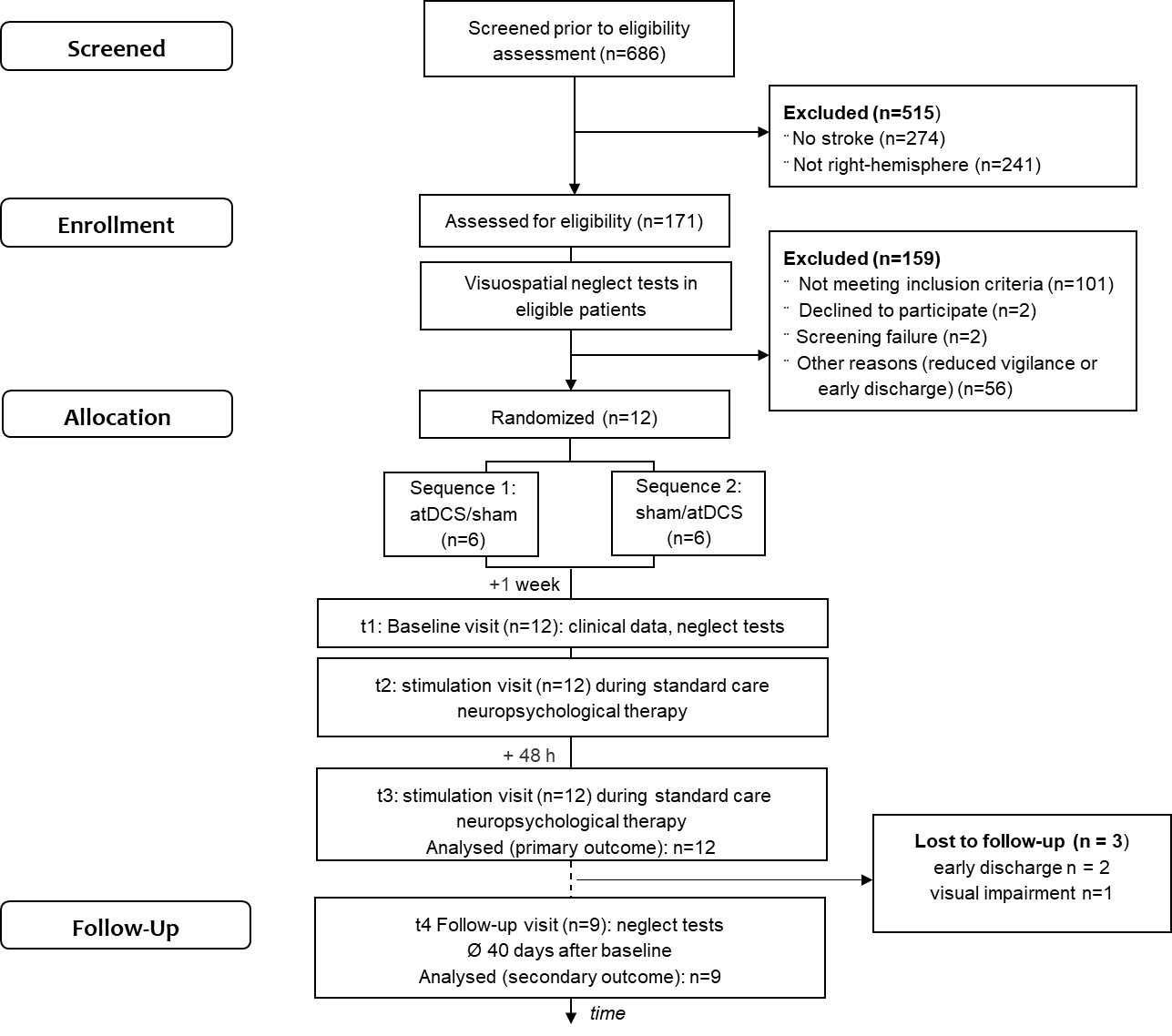
**Figure S1**. Flowchart of cross-over study.

Legend: Six hundred eighty-six patients were pre-screened at the neurological rehabilitation ward of the Kliniken Beelitz GmbH in Brandenburg, Germany, for presence of left-sided visuospatial neglect. Patients with no (n=274) or no right-hemisphere stroke (n=241) were excluded. Remaining patients were screened for inclusion/exclusion criteria. One hundred one patients did not meet the inclusion criteria, 2 patients declined to participate, and 58 were excluded due to screening failure (n=2) or other reasons (n=56) leaving 12 patients diagnosed with left-sided visuospatial neglect for participation in this cross-over study. These patients were randomly assigned to one of two sequences (sequence 1: active transcranial direct current stimulation (atDCS), sham stimulation (stDCS); sequence 2: stDCS, atDCS). After baseline visit (t1) atDCS or stDCS (t2) was applied simultaneous to standard care neuropsychological therapy. After 48 h the other (not yet applied) stimulation condition was conducted (t3). Due to early discharge (n=2) and visual impairment (n=1) only 9 patients performed follow-up tests on the last day of the hospital stay. Thus, 12 patients were analysed for primary objective, and 9 patients for secondary objective.

Table S1: Inclusion and Exclusion criteria

| **Inclusion criteria** |
| --- |
|  |
| Ischemic or hemorraghic stroke in the right hemisphere (confirmed by CT or MRI) |
| Early subacute phase after stroke (defined as 7 to 56 days after stroke onset) |
| Minimum 18 years of age |
| Signs of visuospatial neglect |
| Right-handed |
| Able to understand the scope and content of the trial |
| **Exclusion criteria** |
|  |
| Severe alcohol or narcotic abuse, severe psychiatric disease like depression or psychosis (if not in remission) |
| History of stroke |
| Severe cognitive impairment |
| Visual impairment which cannot be corrected with any optical aid or decreased visual field due to hemianopia. |
| Use of medications primarily affecting the central nervous system including antidepressants, neuroleptics, sedatives, Alpha-1 blockers, psychostimulants |
| Pregnancy |
| Epileptic activity |
| History of severe traumatic brain injury or surgery |
| Pacemaker |
| Participation in any intervention trial |

MRI = magnetic resonance imaging; CT = computer tomography
