# Supplementary material for: Blinding in electric current stimulation in subacute neglect patients with current densities of 0.8 A/m2: a cross-over pilot study
Source: BMC Res Notes. 2021 Jan 25;14:35. doi: 10.1186/s13104-020-05421-7 (PMC7836170; doi:10.1186/s13104-020-05421-7)
Supplement: Supplementary file 3 — Additional file 3. Randomization procedure. [file 13104_2020_5421_MOESM3_ESM.docx]

# Randomization procedure

Randomisation was performed using following R-script (R statistical software, Version 3.4.4):

######################################################

## tDCS Neglekt Pilot 1

## Random Sequenz allocation

## 13.03.2018

######################################################

## convert to rmarkdown for printout

## add Sys.Date() to mark specific time-frame

# set seed to make code reproducable; change seed (default for testing was 123) before study start

set.seed(123)

## add leading zeros and prefix 'KBG-BLIND-'

# sample Patients from 001 - 012

Pat.ID <- sample(1:12, 12, replace = F)

# assign two intervention sequences

Int1 <- c(replicate(6, "Stim"), replicate(6, "Sham"))

Int2 <- c(replicate(6, "Sham"), replicate(6, "Stim"))

# create allocation list

RA.design <- data.frame(Pat.ID, Int1, Int2)

colnames(RA.design) <- c("Pat.ID", "Int1", "Int2")

# export list to file

write.csv(RA.design, file = "Random_allocation_list_Pilot1.csv", row.names = F)
